# Discovery of a Novel Antiviral Effect of the Restriction Factor SPOC1 against Human Cytomegalovirus

**DOI:** 10.3390/v16030363

**Published:** 2024-02-27

**Authors:** Anna K. Kuderna, Anna Reichel, Julia Tillmanns, Maja Class, Myriam Scherer, Thomas Stamminger

**Affiliations:** 1 Institute of Virology, Ulm University Medical Center, 89081 Ulm, Germany; anna.kuderna@uniklinik-ulm.de (A.K.K.); myriam.scherer@uniklinik-ulm.de (M.S.); 2 Instituto de Medicina Molecular João Lobo Antunes, Faculdade de Medicina da Universidade de Lisboa, 1649-028 Lisboa, Portugal; anna.reichel@medicina.ulisboa.pt; 3 Institute of Clinical and Molecular Virology, Friedrich-Alexander-Universität Erlangen-Nürnberg, 91054 Erlangen, Germany; jul.tillmanns@fau.de

**Keywords:** human cytomegalovirus, restriction factor, viral transcription, antiviral, chromatin remodeler, RNA Pol II, dual role

## Abstract

The chromatin-remodeler SPOC1 (PHF13) is a transcriptional co-regulator and has been identified as a restriction factor against various viruses, including human cytomegalovirus (HCMV). For HCMV, SPOC1 was shown to block the onset of immediate-early (IE) gene expression under low multiplicities of infection (MOI). Here, we demonstrate that SPOC1-mediated restriction of IE expression is neutralized by increasing viral titers. Interestingly, our study reveals that SPOC1 exerts an additional antiviral function beyond the IE phase of HCMV replication. Expression of SPOC1 under conditions of high MOI resulted in severely impaired viral DNA replication and viral particle release, which may be attributed to inefficient viral transcription. With the use of click chemistry, the localization of viral DNA was investigated at late time points after infection. Intriguingly, we detected a co-localization of SPOC1, RNA polymerase II S5P and polycomb repressor complex 2 (PRC2) components in close proximity to viral DNA in areas that are hypothesized to harbor viral transcription sites. We further identified the N-terminal domain of SPOC1 to be responsible for interaction with EZH2, a subunit of the PRC2 complex. With this study, we report a novel and potent antiviral function of SPOC1 against HCMV that is efficient even with unrestricted IE gene expression.

## 1. Introduction

Viruses must overcome the barriers of host defense mechanisms to establish a successful infection. The first line of intracellular protection, known as intrinsic immunity, is exerted by so-called restriction factors (RFs), which are constitutively expressed cellular proteins with antiviral functions (reviewed in [1]). A number of RFs against various viruses are known; among them is the cellular protein SPOC1 (survival time-associated PHD (plant homeodomain) in ovarian cancer 1), also called PHF13 (PHD finger 13) [2,3,4].

This highly conserved protein was firstly described in 2005 in the context of ovarian carcinoma, where high expression levels were demonstrated to correlate with poor survival prognosis [5]. Under physiological conditions, enhanced expression of SPOC1 is reported in fast-proliferating cell types [5]. Bördlein et al. showed in 2011 that the protein is essential for stem-cell differentiation in testis and spermatogenesis [6]. Furthermore, SPOC1 is important for regulating the DNA damage response. Here, it is recruited to DNA double-strand breaks, influences DNA compaction factors and favors homologous recombination over non-homologous end joining [7]. SPOC1 is located in chromosomal region 1p36.3, and the encoded protein is 300 amino acids in length. It comprises an N-terminal domain (NTD), two PEST domains and a PHD domain and harbors a nuclear localization signal [8].

The PEST domains contribute via their peptide sequences, consisting of proline (P), glutamic acid (E), serine (S) and threonine (T), to the instability of soluble SPOC1. In contrast, the protein gains stability when bound to chromatin [8]. This chromatin association is either mediated by the PHD domain or by a centrally located region of SPOC1. SPOC1 is able to directly bind chromatin via H3K4me2/3 interactions and indirectly via the polycomb repressive complex 2 (PRC2) and RNA polymerase II (RNAP II) [9]. SPOC1 targets genes that are transcriptionally more active and is classified as a H3K4me2/3 reader. Chung et al. showed in 2016 that a depletion of SPOC1 leads to the disturbance of interactions between PRC2, the serine 5 phosphorylated form of RNAP II (RNAPII S5P) and H3K4me3/H3K27me, which subsequently leads to up- or downregulation of various genes [10]. SPOC1 was therefore concluded to function as a transcriptional regulator that favors either activation or repression of specific genes. This transcriptional regulation was found to be dependent on the specific chromatin landscape of the respective gene. In the presence of histone modifications H3K4me2/3 and H3K27me3 as well as PRC2 components and RNAP II S5P, SPOC1-bound genes were found to be inaccessible and repressed. In contrast, another group of SPOC1-bound genes, which displayed a preponderance of H3K4me2/3, RNAP II S2P and S5P, was found to be actively expressed [10]. SPOC1 functions as a scaffold stabilizing PRC2 and RNAPII S5P. Upon SPOC1 depletion, an upregulation of PRC2-repressed genes was reported [10]. Furthermore, binding of SPOC1 has been shown to shift histone modifications towards repressive marks via recruiting several heterochromatin-inducing factors, such as histone methyltransferases SETDB1 and G9A [7].

In the context of viral infection, SPOC1 has been identified as an RF against adenovirus (Ad) [2], human immunodeficiency virus 1 (HIV-1) [3] and human cytomegalovirus (HCMV) [4]. HCMV, a highly adapted β-herpesvirus, remains mostly asymptomatic in immunocompetent individuals but can lead to severe and life-threatening diseases in immunocompromised patients (reviewed in [11]). During lytic infection, viral gene expression occurs in three sequential phases. In the immediate-early (IE) phase, the virus antagonizes cellular barriers and establishes an optimal cellular environment for the onset of the early (E) phase, which prepares for viral DNA replication. During the late (L) phase, DNA replication takes place, and novel viral particles are assembled (reviewed in [12]). The main regulatory element of the IE phase and all subsequent steps of viral infection is the major immediate early promoter (MIEP). This promoter drives expression of the two main IE gene products, IE1 and IE2. These two proteins, which are encoded by spliced RNAs, act as important effector proteins, regulating further downstream events of HCMV infection (reviewed in [13]). SPOC1 has been demonstrated to associate with the MIEP during the very first hours of HCMV infection, which correlates with a block of IE gene expression, probably via the recruitment of heterochromatin building factors [4]. It has been shown that SPOC1-mediated restriction only takes place when SPOC1 is expressed during the start of infection. So far, a role of SPOC1 during later times of lytic replication has not been investigated. Interestingly, for HIV-1, SPOC1 has been reported to play a dual role during the viral life cycle [3]. When SPOC1 levels were enhanced prior to viral integration, it favored HIV-1 integration, while SPOC1 overexpression after integration displayed an antiviral role via suppression of viral gene expression.

Here, we were interested to answer the question of whether SPOC1 exerts additional functions during later phases of the HCMV replication cycle. Not only did we find that this cellular protein acts as a cellular restriction factor of viral IE gene expression, but we observed a strong negative impact of SPOC1 on viral particle release. Under high MOI conditions that ensured an undisturbed IE phase in SPOC1-expressing cells, we found a severely impaired viral DNA replication. SPOC1 potently blocked viral gene transcription, resulting in diminished protein expression. Furthermore, we detected a co-localization of SPOC1 with PRC2 components and RNAP II S5P in close proximity to viral DNA at late times of replication. Finally, we confirmed the interaction of SPOC1 with the PRC2 component EZH2 and found the N-terminal domain of SPOC1 to mediate this interaction. Based on these data we conclude that SPOC1 not only represses the MIEP directly after the onset of infection but also mediates a second defense layer in cells with incomplete shutdown of the IE phase via repressing viral early and late gene transcription.

## 2. Materials and Methods

### 2.1. Plasmids and Cloning

The coding sequence of SPOC1 was cloned into the pHM971 plasmid (pcDNA3-based expression vector with FLAG-tag, described in [14] with oligonucleotides SPOC1_BamHI_fw and SPOC1_EcoRI_rev, resulting in N-terminally FLAG-tagged SPOC1 WT [pHM4299]). pHM971 without SPOC1 served as the negative control. Furthermore, deletion mutants of SPOC1 were generated by amplification of the relevant SPOC1 sequences with the same forward primer and SPOC1_231.EcoRI_rv (aa 1-231) or SPOC1_150.EcoRI_rv (aa 1-150) and cloning into the same plasmid. N-terminally FLAG-tagged SPOC1 deletion mutant aa 64-300 (pF1017) was kindly provided by Andreas Winterpacht (Erlangen, Germany), as well as a pcDNA3.1-based expression vector for EZH2 (pF1071). For primer sequences, see Table S1.

### 2.2. Cells and Transfection

Primary human foreskin fibroblasts (HFFs) were cultivated in Eagle’s minimal essential medium (Gibco, Thermo Fisher Scientific Inc., Waltham, MA, USA) containing 7% fetal calf serum (FCS) (Sigma-Aldrich, Merck KGaA, Darmstadt, Germany), 1% GlutaMAX (Gibco, Thermo Fisher Scientific Inc., Waltham, MA, USA), and penicillin-streptomycin (Sigma-Aldrich, Merck KGaA, Darmstadt, Germany) at 37 °C and 5% CO_2_. The stable SPOC1-expressing cells (HFF/SPOC1) and the respective control cells (HFF/Ctrl) as well as SPOC1-inducible HFFs (HFF/ind.SPOC1) were described in [4]. HFF/mCherry-SPOC1 cells, which were utilized for immunofluorescence experiments, were generated as follows: the SPOC1 coding sequence was cloned into the expression plasmid pLKO-EF1-α-mCherry and used for generation of replication-deficient lentiviral particles (as previously described in [4]). The lentiviral supernatant was then utilized for transduction of HFFs, which resulted in HFF/mCherry-SPOC1. Additionally, a control cell line HFF/mCherry was established [15]. For selection, the medium was supplemented with 500 μg/mL geneticin (InvivoGen, Toulouse, France). Cells displayed a transduction efficiency of 70% [15]. HEK293T cells were cultivated in Dulbecco’s minimal essential medium (DMEM) supplemented with glutamine (Gibco, Thermo Fisher Scientific Inc., Waltham, MA, USA), 10% FCS (Sigma-Aldrich, Merck KGaA, Darmstadt, Germany) and penicillin-streptomycin (Sigma-Aldrich, Merck KGaA, Darmstadt, Germany).

### 2.3. Infection and Virus Stock Titration

On day one, 3 × 10^5^ HFF/well were seeded in 6 wells and incubated for two days to ensure complete density. On day four, cells were infected with either HCMV TB40/E wildtype (WT) [16] or AD169 WT [17], with multiplicities of infection (MOI) between 0.01 and 3. Cells were provided with fresh medium at 1.5 hpi. Virus titration, based on IE protein-forming units (IEUs), was performed as previously described in [18].

### 2.4. Western Blotting and Quantification

Whole cell lysates from harvested cells were mixed with 4× sodium dodecyl sulfate-polyacrylamide gel electrophoresis (SDS-PAGE) loading buffer and incubated at 95 °C for 10 min prior to 1 min of sonification (Q700 Sonicator, QSonica, Newton, MA, USA). The samples were separated on 10% or 12% SDS-polyacrylamide gels and afterwards transferred onto PVDF membranes (BioRad, Feldkirchen, Germany). Using a FUSION FX7 imaging system (Vilber Lourmat, Eberhardzell, Germany), proteins were detected via chemiluminescence. The antibodies that were used for infection experiments were anti-β-actin AC15 (Sigma-Aldrich, Merck KGaA, Darmstadt, Germany), anti-SPOC1 m45/6F6 (kindly provided by Sabrina Schreiner, Freiburg, Germany) [8], anti-IE1 p63-27 [19], anti-IE2 pAB178 [14], anti-UL44 BS510 (kindly provided by B. Plachter, Mainz, Germany), anti-MCP 28-4 [20] and anti-pp28 41-18 [21]. Anti-EZH2 (Cell Signaling Technology, Frankfurt am Main, Germany) and anti-FLAG 1804 (Sigma-Aldrich, Merck KGaA, Darmstadt, Germany) antibodies were used for CoIP experiments. Horseradish peroxidase-conjugated anti-rat, anti-mouse and anti-rabbit secondary antibodies for Western blot analysis were purchased from Dianova (Hamburg, Germany). For quantification, the signal intensities of each band on one membrane were measured using the Image Lab software (Bio-Rad, Feldkirchen, Germany) and normalized to beta-actin stained on the same membrane.

### 2.5. Replication and Release Assays

Quantification of intra- and extracellular viral DNA was performed via quantitative TaqMan real-time PCR using infected cells and their supernatants at 96 hpi as described elsewhere [22]. The utilized oligonucleotides were CMV/IE1 plus the regarding probe MIE FAM/TAMRA as well as Albumin primers and Alb FAM/TAMRA. For primer sequences, see Table S1.

### 2.6. RNA Isolation and Quantitative SYBR Green Reverse Transcription-PCR (qRT-PCR)

RNA was isolated from infected cells, cDNA was synthesized and used for qRT-PCR using the AriaMx Real-Time PCR System (Agilent, Santa Clara, CA, USA) as previously described [23]. Primer pairs for GAPDH, UL123 (IE1), UL122 (IE2) [24], US3 [25], UL44 [26], UL82 (pp71) [27], UL86 (MCP) [26] and UL99 (pp28) [25] were used. For primer sequences, see Table S1.

### 2.7. Indirect Immunofluorescence

Indirect immunofluorescence analysis was performed as described elsewhere [28]. For investigation of later stages of HCMV infection, the cells were treated with 2 mg/mL γ-globulins from human blood (Sigma-Aldrich, Merck KGaA, Darmstadt, Germany) prior to incubation with primary antibodies (see Western Blotting and Quantification). Additionally, anti-SUZ12 (Cell Signaling Technology, Frankfurt am Main, Germany), anti-RNA Pol II 4H8 (Abcam, Berlin, Germany) and anti-RNA Pol II 8WG16 (Covance, Princeton, NJ, USA) were used as primary antibodies. Secondary antibodies utilized for indirect immunofluorescence were combinations of mouse/rat/rabbit Alexa-488/-555 or -647 (Invitrogen, Carlsbad, CA, USA).

### 2.8. Labelling of Viral DNA

For direct visualization of viral DNA, either 2′-desoxy-2′-fluor-5-ethinyluridin (F-Ara-EdU) (Sigma-Aldrich, Merck KGaA, Darmstadt, Germany) [15] or 5-ethynyl-20-deoxy-cytidine (EdC) (Sigma-Aldrich, Merck KGaA, Darmstadt, Germany) was used. Cells were seeded on coverslips followed by HCMV infection. F-Ara-EdU (50 µM) or EdC (1 µM) was added at 72 hpi. At 96 hpi, the cells treated with F-Ara-EdU were fixed, permeabilized and stained as described above. Click chemistry (copper (I)-catalyzed azide–alkyne cycloaddition) was performed according to [29], with 10 μM Alexa-555-azide in the labelling solution, for 2 h at RT. After three washing steps, the coverslips were sealed as described elsewhere [28]. When working with the EdC-treated cells, a quenching step (50 mM glycine and 50 mM NH_4_Cl in 0.01 M PBS; for 5 min at RT) was performed prior to membrane permeabilization. After staining with primary and secondary antibodies (for antibodies, see Section 2.4, Western Blotting and Quantification, and Section 2.7, Indirect Immunofluorescence), cells were washed three times and incubated in the dark in a labelling solution containing 0.01 M PBS, 4.15 mM A488 azide (Thermo Fisher Scientific Inc., Waltham, MA, USA), 1 M Na-ascorbate, 100 mM CuSO_4_, 1 M aminoguanidine and 100 mM THPTA at RT. Coverslips were prepared for detection as described above.

### 2.9. Co-Immunoprecipitation

Co-Immunoprecipitation (CoIP) was performed with HEK293T cells co-transfected with an EZH2-expressing plasmid and either a control plasmid expressing only FLAG or different FLAG-tagged deletion mutants of SPOC1 (see Plasmids and Cloning). At 48 h post transfection, the cells were harvested, and CoIP was performed as described in [28] but without sonification. Anti-Flag Magnetic Beads (MedChemExpress, Monmouth Junction, NJ, USA) were used to pull down FLAG-SPOC1.

## 3. Results

### 3.1. HCMV Overcomes SPOC1-Mediated Immediate-Early Repression with Increasing Viral Doses

**Figure 1:**
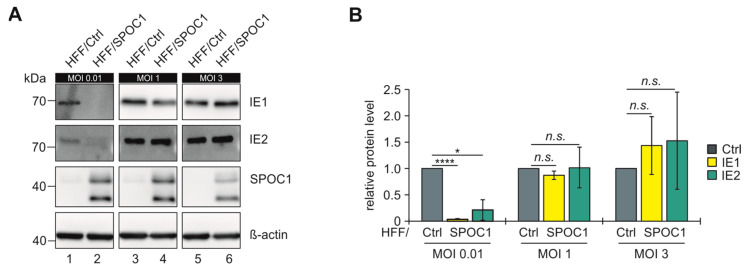
Increasing HCMV doses are able to antagonize SPOC1-mediated repression of IE1 and IE2 expression. (**A**) The 24 hpi lysates of HCMV-infected control fibroblasts (HFF/Ctrl) and fibroblasts expressing SPOC1 (HFF/SPOC1) infected with HCMV TB40/E at MOIs of 0.01 (lanes 1 and 2), 1 (lanes 3 and 4) and 3 (lanes 5 and 6) were investigated by Western blotting. Expression levels of viral immediate-early proteins IE1 and IE2, β-actin and SPOC1 were analyzed. (**B**) Quantification of IE1 and IE2 signal intensities normalized to β-actin levels in HFF/SPOC1 relative to normalized IE1 and IE2 levels in HFF/Ctrl of three independent experiments. Statistical analysis was performed using Student’s *t*-test (one sample, two-tailed); **** *p* < 0.0001, * *p* < 0.05, *n.s.* = not significant.

SPOC1 is a potent restriction factor during the immediate-early phase of HCMV infection, but so far, it remains unknown whether the protein plays an additional role during later phases of lytic replication. To investigate this, we established conditions to circumvent SPOC1-mediated IE repression. One hallmark of restriction factors is that a higher viral load enables the viruses to overcome the respective restrictive effects [30,31]. Therefore, we infected SPOC1-expressing fibroblasts (HFF/SPOC1) or control cells (HFF/Ctrl) with increasing MOIs (0.01, 1 and 3) and analyzed the levels of the IE1 and IE2 effector proteins at 24 hpi as a marker for an effective rescue of SPOC1-mediated repression of the major immediate-early promoter (MIEP). We observed that while SPOC1 efficiently suppressed IE protein expression at an MOI of 0.01, higher MOIs of 1 or 3 led to comparable IE protein levels after infection of HFF/Ctrl or HFF/SPOC1, respectively (Figure 1, panels A and B). Based on these results we conducted all subsequent experiments at an MOI of 1 or higher to ensure a comparable start of infection in both cell populations. This allowed us to be sure that viral replication differences in SPOC1-expressing cells can be attributed to effects exerted by SPOC1 during later phases of infection.

### 3.2. HCMV DNA Replication and Viral Particle Release Are Strongly Restricted upon SPOC1 Expression

**Figure 2:**
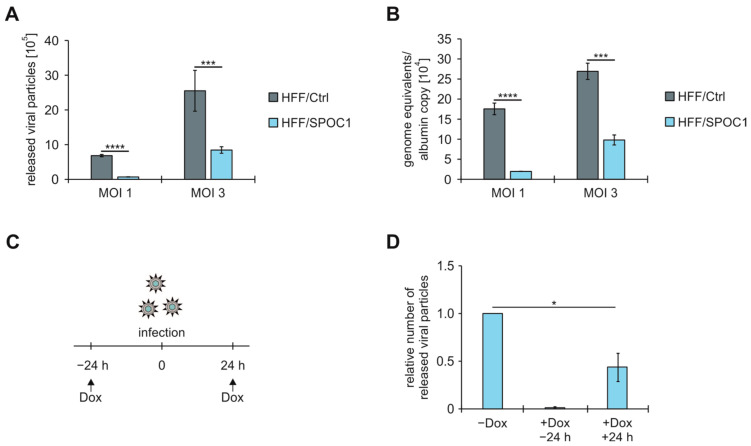
SPOC1 expression negatively affects HCMV DNA replication and viral particle release. HFF/Ctrl and HFF/SPOC1 were infected in triplicate with HCMV strain TB40/E at an MOI of 1 or 3. (**A**) At 96 hpi, supernatants were analyzed for viral genome equivalents via qPCR. (**B**) At 96 hpi, intracellular DNA was isolated and HCMV genome equivalents were quantified via qPCR and normalized to albumin copy numbers. One out of three independent experiments is shown. Statistical analysis was performed using Student’s *t*-test (unpaired, two-tailed); *** *p* < 0.001, **** *p* < 0.0001. (**C**) Experimental set-up: Doxycycline (Dox)-inducible HFF/SPOC1 cells were either treated with Dox 24 h prior to or 24 h post infection with AD169 at MOI 0.1. (**D**) Dox-inducible HFF/SPOC1 was infected with AD169, at MOI 0.1, in triplicate. The infected cells were either left untreated or treated with Dox 24 h prior to or 24 h post infection (**C**). At 96 hpi, the supernatant was harvested and analyzed for viral genome equivalents via qPCR. One out of two experiments is shown. Statistical analysis was performed utilizing the one sample *t*-test; * *p* < 0.05.

With the use of high doses of HCMV, we established conditions that ensured an unbothered IE phase after infection of SPOC1-expressing cells. This enabled us to investigate the overall impact of SPOC1 on HCMV independent of its already described function as a restriction factor of the IE phase. To test whether SPOC1 exerts effects during later phases of HCMV replication, we analyzed the release of viral particles after one complete replication cycle under conditions of high MOI. For that purpose, HFF/Ctrl and HFF/SPOC1 were infected with TB40/E at MOIs of 1 and 3. At 96 hpi, supernatants were harvested and viral genome equivalents were determined via qPCR (Figure 2A). After infection of HFF/SPOC1, almost no viral particles were released at MOI 1, and significantly fewer particles could be detected at MOI 3 in comparison to control cells (HFF/Ctrl). In order to answer the question of whether the antiviral function of SPOC1 already affects viral DNA replication, we harvested cells that were infected under the same conditions followed by extraction of DNA. The obtained DNA was analyzed for viral genome equivalents and normalized to albumin copy numbers (Figure 2B). This revealed a strong decrease of viral DNA replication in the presence of SPOC1.

To further confirm this, we used fibroblasts with doxycycline-inducible SPOC1 expression. Reichel et al. showed that expression of SPOC1 24 h prior to infection led to significantly lower IE1 protein levels, while SPOC1 expression at 8 hpi was no longer able to repress IE1 [4]. Based on that finding, we infected our cells with AD169 and expressed SPOC1 either 24 h prior to or 24 h post infection, as the IE phase of HCMV should be completed at 24 hpi. When we investigated the viral release after 96 h, we found almost no released particles when SPOC1 was expressed prior to infection. Furthermore, and consistent with our observations using stable HFFs infected at high MOIs, we found that SPOC1 expression after the IE phase of HCMV infection led to a significant downregulation of viral particle release. From those results, we concluded that the cellular protein SPOC1 plays an additional antiviral role against HCMV during later phases of the replication cycle that seems to be virus strain- and MOI-independent.

### 3.3. SPOC1 Leads to Reduced Early and Late Viral Protein Expression

**Figure 3:**
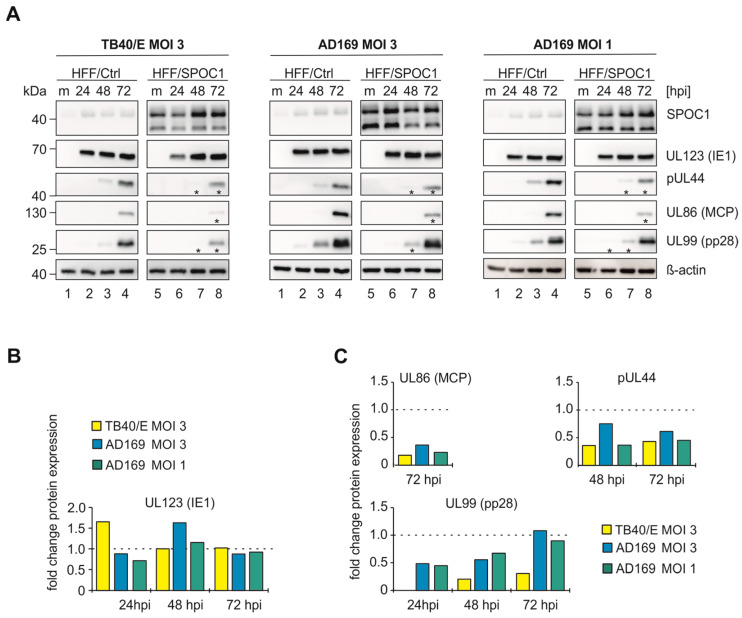
SPOC1 leads to lower expression levels of viral early and late proteins. (**A**) Lysates of mock-infected (m) or TB40/E- (MOI of 3) or AD169-infected (MOI of 3 and 1) HFF/Ctrl and HFF/SPOC1 cells were analyzed at 24 to 72 hpi by separation on a 10 % polyacrylamide gel followed by Western blot detection of indicated proteins. Expression kinetics of viral immediate-early protein IE1, viral early protein pUL44 and viral late proteins pp28 and MCP were investigated. Asterisks (*) highlight the protein bands that are attenuated upon SPOC1 expression. (**B**) Quantification of IE1 levels in HFF/SPOC1 normalized to β-actin are depicted as fold change of the normalized IE1 level of HFF/Ctrl (indicated by dashed line at y = 1). (**C**) Quantification of early and late viral proteins of HFF/SPOC1 normalized to β-actin are depicted as fold change of the regarding normalized protein levels of HFF/Ctrl (indicated by dashed line at y = 1).

To narrow down the time point of impact of SPOC1 during HCMV infection, we next analyzed the expression of different viral early and late proteins at 24 to 72 hpi via Western blotting. In order to exclude strain-specific effects, we used the HCMV strains TB40/E and AD169 at MOIs of 1 or 3 (Figure 3). Again, we ensured that IE1 protein expression levels were comparable between both cell populations in each setup (Figure 3B). For all tested viral early and late proteins, a diminished expression was observed after infection of HFF/SPOC1 cells compared to the control cells (Figure 3A). This was true for all infection conditions, with the strongest effects observed for TB40/E infection. The signal intensities for each viral protein were quantified and normalized to β-actin levels (Figure 3C).

The overall downregulation of all tested viral proteins indicates that SPOC1 might have a general negative impact on the early and late phase of lytic HCMV infection or negatively influence an early event that in turn regulates all following steps. As similar tendencies for all conditions were observed, and already an MOI of 1 led to clearly visible changes in protein levels, the following experiments were also conducted under these conditions when more practicable.

### 3.4. SPOC1 Blocks Transcription of Viral Early and Late Genes

So far, we have observed that, independent of the viral immediate-early phase, SPOC1 expression leads to impaired viral DNA replication and particle release and a downregulation of the expression of viral proteins. As SPOC1 is known to act as a transcriptional regulator [10], we next asked whether the observed diminished viral protein levels might be due to the repressive effects of SPOC1 on viral transcription exerted during the early and late phase of the replication cycle.

**Figure 4:**
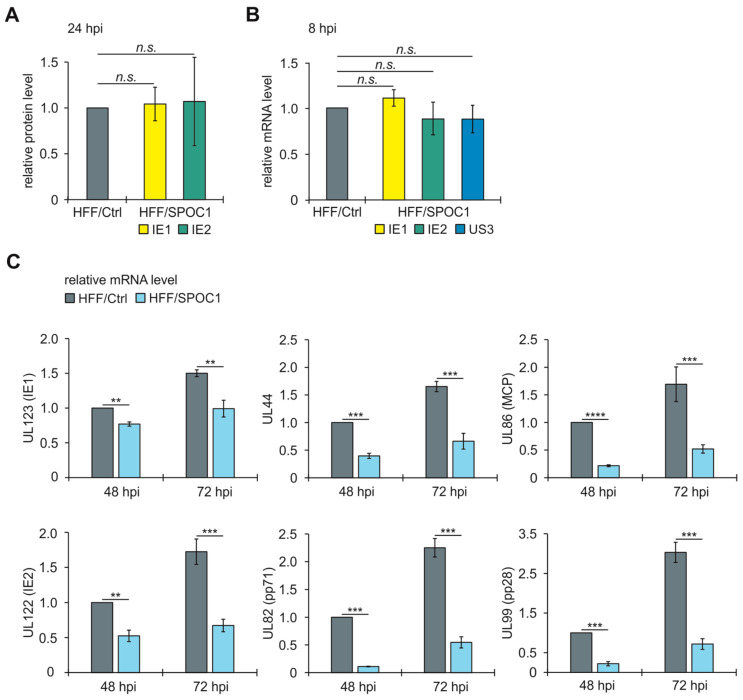
Impaired transcription of viral early and late genes in HFF/SPOC1 cells at late times in the replicative cycle. HFF/Ctrl and HFF/SPOC1 cells were infected with AD169 at an MOI of 1. (**A**) The 24 hpi IE1 and IE2 protein levels were analyzed via SDS PAGE and Western blotting. The relative intensity values were normalized to β-actin. Quantification of three independent experiments is shown. Statistical analysis was performed using Student’s *t*-test (one sample, two-tailed); *n.s.* = not significant. (**B**) IE1, IE2 and US3 transcript levels normalized to GAPDH were evaluated at 8 hpi using qPCR. Shown are the mean values of triplicates of one out of two experiments. Statistical analysis was performed using Student’s *t*-test (one sample, two-tailed). (**C**) At 48 and 72 hpi, total cellular RNA was isolated, cDNA was synthesized and viral mRNA levels of two immediate-early, early and late genes were quantified via qPCR, respectively. Shown are the mean values of triplicates of one out of three experiments. Statistical analysis was performed with Student’s *t*-test (unpaired, two-tailed); ** *p* < 0.01, *** *p* < 0.001, **** *p* < 0.0001.

HFF/Ctrl and HFF/SPOC1 were infected with AD169 at an MOI of 1. We firstly ensured comparable IE protein expression levels at 24 hpi in both cell lines via Western blotting and quantification of the respective signals (Figure 4A). As a further control, IE1 and IE2 mRNA levels were investigated at 8 hpi, as the peak of IE transcription is described to occur during the very early hours after infection [32,33]. Additionally, we included the IE gene US3, which is transcribed under control of a different HCMV IE promoter (Figure 4B) [34]. Infected cells were harvested, followed by RNA isolation, cDNA synthesis and SYBRgreen qPCR (Figure 4B). As shown in Figure 4, panel B, the transcript levels of IE1, IE2 and US3 were comparable in HFF/Ctrl and HFF/SPOC1 at 8 hpi. At 48 and 72 hpi, when viral early and late transcription takes place, we used the same approach with primers for either immediate-early (UL123 [IE1], UL122 [IE2]), early (UL44, UL82 [pp71]) or late viral genes (UL86 [MCP], UL99 [pp28]). All mRNA levels were normalized to transcription of the housekeeping gene GAPDH.

Consistent with diminished protein levels (Figure 3), we observed a strong and significant transcriptional downregulation of each tested gene at late times of the replicative cycle in SPOC1-expressing cells (Figure 4C). Interestingly, this effect was also true for IE transcripts, indicating the possibility that SPOC1, despite an unaffected IE phase, affects the transcription of viral genes of all temporal classes at late times of the replicative cycle.

### 3.5. SPOC1 Localizes in Close Proximity to Viral DNA during Later Time Points of the Replicative Cycle

**Figure 5:**
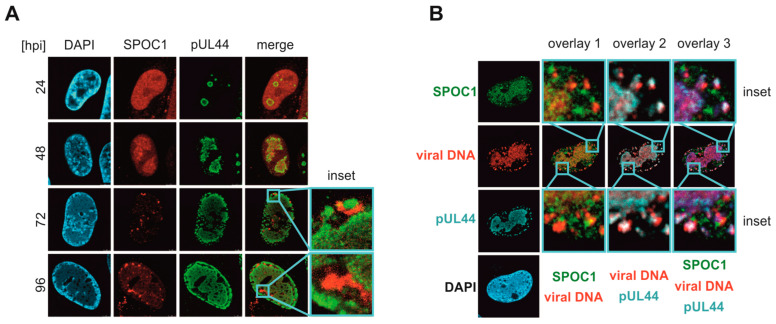
SPOC1 localization during the time course of HCMV infection. (**A**) HFF/mCherry-SPOC1 cells were infected with AD169 at MOI 1 and fixed at the indicated time points. An antibody against pUL44 was used in combination with the secondary antibody Alexa-488. DAPI staining was used to visualize the nucleus. (**B**) At 72 hpi, AD169-infected HFF/SPOC1 (MOI of 1) was treated with F-Ara-EdU prior to fixation at 96 hpi. Samples were stained for SPOC1 (secondary antibody: Alexa-488) as well as with an antibody against pUL44 in combination with the Alexa-647 antibody. Click chemistry was performed to visualize viral DNA (modified from [15]).

It has been shown that the binding of SPOC1 to specific target genes can lead to chromatin compaction and repression [7]. Since we observed downregulation of viral gene expression, not only for protein but even stronger for transcriptional levels, we were interested to investigate a potential association of SPOC1 with viral DNA. For that, the localization of SPOC1 during the time course of the HCMV lytic replication cycle was analyzed using HFFs that express SPOC1 fused to the autofluorescent protein mCherry. Interestingly, at 48 h after infection with AD169 (MOI 1), SPOC1 accumulated in viral replication centers (VRCs) marked by pUL44, while during later stages of infection, SPOC1 was redistributed and could be detected in distinct clusters at the peripheries of the VRCs in close proximity to pUL44 (Figure 5A). In contrast, the control cell population HFF/mCherry showed no mCherry signal in the cell nucleus at any time point of infection (Figure S1), underlining the specificity of the observed mCherry-SPOC1 localization.

To further evaluate our hypothesis that SPOC1 might associate with viral DNA, we tested whether the observed protein accumulations co-localize with viral DNA. For that purpose, F-ara-EdU was utilized for dynamic metabolic labeling of the viral DNA. SPOC1-expressing HFFs were infected (AD169, MOI of 1), and 72 hpi F-ara-EdU was added. At 96 hpi, the cells were fixed and additionally stained for SPOC1 and pUL44. Click chemistry (copper (I)-catalyzed azide–alkyne cycloaddition) was performed to visualize viral DNA. The VRCs in which active viral replication took place were successfully marked by F-Ara-EdU (Figure 5B; Figure S2). In line with our previous results, we observed an overall negative correlation between high expression of SPOC1 and the presence of viral DNA in immunofluorescence analysis, emphasizing our findings of downregulated (p)UL44 and impaired viral DNA synthesis in SPOC1-expressing cell populations (Figure S2). Additionally, a dot-shaped pattern of viral DNA at the nuclear rim was detected that co-localized with pUL44 (Figure 5B). The SPOC1 signal was found to be located in the VRCs and at the nuclear rim, in close proximity to pUL44 and viral DNA, indicating a potential association of SPOC1 with viral DNA.

### 3.6. SPOC1 Interacts and co-Localizes with PRC2 Adjacent to Viral DNA

**Figure 6:**
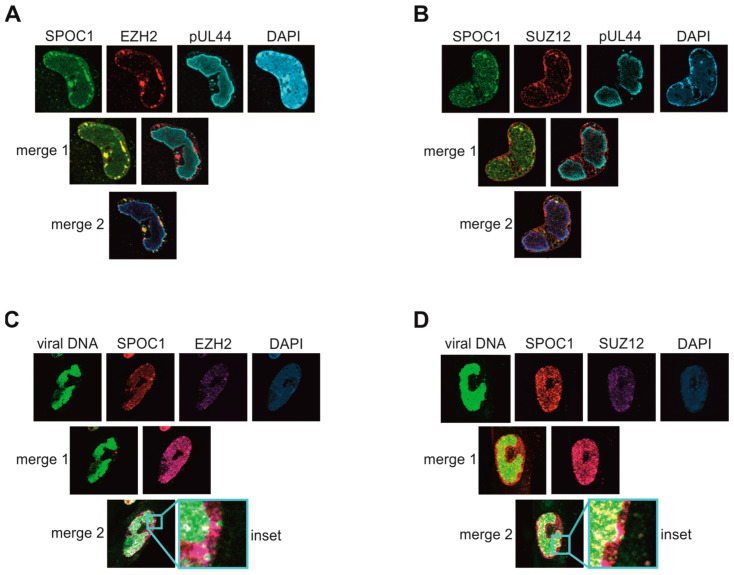
SPOC1 co-localizes with PRC2 components close to viral DNA. HFF/SPOC1 was infected with AD169 at an MOI of 1. At 96 hpi, the cells were fixed and treated with antibodies directed against SPOC1, pUL44 and either (**A**) EZH2 or (**B**) SUZ12. As secondary antibodies, Alexa-488 (SPOC1) and a combination of either mouse or rabbit Alexa-555 and mouse or rabbit Alexa-647 were used. DAPI signals visualize the nucleus (modified from [15]). (**C**,**D**) At 72 hpi, EdC was added to SPOC1-expressing cells prior to fixation at 96 hpi. The same antibodies against EZH2 (**C**) or SUZ12 (**D**) were used as for (**A**) and (**B**); viral DNA was visualized by click chemistry. Nuclei were visualized by DAPI staining. Merge images were created from the two images above.

One known interaction partner of SPOC1 is the polycomb repressive complex 2 (PRC2) [10], consisting of the four subunits SUZ12, EED, EZH1 and EZH2. The complex plays important roles for gene silencing and has histone methyltransferase activity [35]. Furthermore, the combination of PRC2, RNAP II S5P and SPOC1 was found to be localized at repressed genes [10]. In the context of HCMV infection, PRC2 was described to play a dual role. While on the one hand, this protein complex is able to silence HCMV genomes to establish and maintain latency [36], it has also been shown to exert a non-canonical function that supports lytic viral DNA replication [37]. With localization studies of PRC2, SPOC1 and viral DNA markers, we aimed to investigate the possible involvement of PRC2 components in mitigating viral transcription during lytic HCMV infection. Viral DNA was visualized either via click labeling or by staining for the viral replication marker pUL44 (Figure 6). HFF/SPOC1 cells were infected with AD169 at MOI 1, fixed after 96 hpi and immunostained for SPOC1, pUL44 and either EZH2 (Figure 6A) or SUZ12 (Figure 6B). We observed a strong co-localization of SPOC1 and both components of the PRC2 complex in dotted structures at the nuclear rim. When viral DNA was visualized, it appeared in the VRCs but also in small dots excluded from the viral replication centers and in close proximity to SPOC1 and PRC2 (Figure 6, panels C and D).

**Figure 7:**
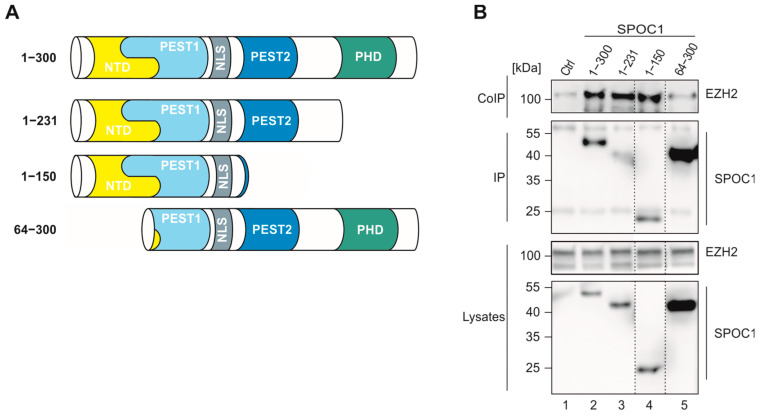
Interaction between SPOC1 deletion mutants and EZH2. (**A**) Schematic representation of the generated FLAG-SPOC1 deletion mutants with indicated amino acid sequence. (**B**) HEK293T cells were co-transfected with an empty control plasmid or FLAG-SPOC1 deletion mutants together with an EZH2-expressing plasmid. FLAG-SPOC1 was precipitated, and lysate controls as well as immuno-precipitated (IP) samples were analyzed via Western blotting. SPOC1 was visualized using an anti-FLAG antibody. CoIP = Co-Immunoprecipitaton.

To further confirm this finding and to narrow down the potential protein interaction interface of SPOC1 and EZH2, various FLAG-tagged SPOC1 mutants were generated, each lacking specific domains of the protein (Figure 7A). The FLAG-SPOC1 plasmids or an empty control plasmid was co-transfected with an EZH2-expressing plasmid in HEK293T cells. SPOC1 was precipitated using an antibody against the FLAG-tag, and a potential interaction of the two proteins was analyzed via Western blotting and staining for EZH2 (Figure 7B). Despite a very strong expression of the deletion mutant SPOC1 64–300 in lysate control and immunoprecipitation (IP), only a faint EZH2 band was visible in the co-immunoprecipitation (CoIP), which is comparable to the control sample in lane 1. As all other SPOC1 mutants, even mutants exhibiting very low expression levels yielded strong EZH2 signals after co-precipitation; we concluded that the N-terminal domain of SPOC1 might be crucial for the interaction with the PRC2 component EZH2.

### 3.7. Co-Localization of SPOC1 and RNA Pol II during Late Stages of Infection

**Figure 8:**
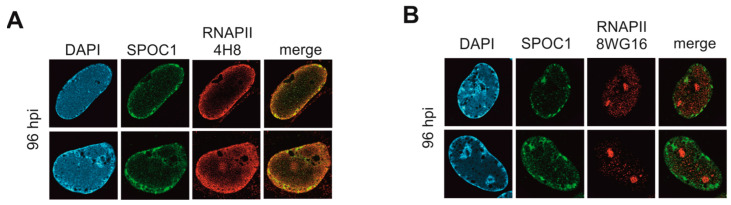
Co-localization of SPOC1 with RNA pol II S5P. HFF/SPOC1 was infected with AD169 (MOI 1) and fixed at 96 hpi. Indirect immunostaining was performed using antibodies against SPOC1 and (**A**) 4H8 antibody detecting specifically Ser5 phosphorylated RNA pol II (modified from [15]) or (**B**) 8WG16 antibody to mark general RNA Pol II localization. DAPI was used to stain cell nuclei.

So far, we have provided indications for interaction and co-localization of SPOC1 and PRC2 components with viral DNA that is present in dotted structures at the nuclear rim but not inside VRCs. As already mentioned, the specific combination of various histone modifications, PRC2, SPOC1 and particular forms of phosphorylated RNAP II influences the mode of action of SPOC1 and thereby decides between activation and repression of target genes [10]. Interestingly, in the context of HCMV infection, Tamrakar and colleagues demonstrated that only the RNAP II S5P is excluded from VRCs but is localized in accumulations at the nuclear rim. In contrast, when using antibodies detecting all forms of RNAP II, they observed signals mainly in VRCs. They concluded that active viral transcription takes place in areas surrounding the replication centers and is physically separated from viral DNA synthesis [38]. Therefore, we decided to analyze the RNAP II localization during HCMV infection in HFF/SPOC1 cells. AD169-infected cells were fixed at 96 hpi and stained for SPOC1 and RNAP II S5P. Consistent with Tamrakar et al., this staining resulted in signals at the nuclear rim that are distinct from viral replication centers. Additionally, we found a clear co-localization of SPOC1 and RNAP II S5P (Figure 8A).

In contrast, when we used the antibody 8WG16, which recognizes unmodified RNAP II, we observed a shift of the signal towards a rather dispersed nuclear pattern with accumulations inside of nucleoli (Figure 8B). Thus, as proposed by Tamrakar and colleagues, it is highly suggestive that the punctuate accumulations at the nuclear rim of infected cells represent regions of ongoing viral transcription [38]. Since we observed a co-localization of viral DNA with RNAP II S5P, PRC2 components and SPOC1 at these punctuate structures, we hypothesize that this induces a repressive chromatin landscape that negatively affects the expression of viral genes at late times of the HCMV replicative cycle.

## 4. Discussion

SPOC1 has been shown to potently repress the expression of HCMV immediate-early genes via binding and blocking the MIEP, thereby exerting a strong antiviral effect during the IE phase and affecting all subsequent steps of infection [4]. This antiviral effect was shown to be MOI-dependent [4], which is a phenomenon that can be observed for many restriction factors [30,31]. With this study, we confirm that the known antiviral function of SPOC1 on HCMV IE gene expression is only effective under low MOI conditions, while high viral loads rescue the initiation of lytic HCMV infection and overcome the first antiviral defense mediated by SPOC1 (Figure 1).

In addition, we report on a second antiviral role of SPOC1 that takes place at a later stage of infection. Our data show that the expression of SPOC1 in the context of a rescued/unaffected IE phase (ensured either by usage of high viral doses or by inducible SPOC1-expressing cell systems in combination with low viral loads) heavily impairs viral DNA replication and particle release (Figure 2). While the effects observed under conditions of high MOI are quite strong, they are less pronounced when investigated with the inducible cells. Reichel et al. showed that SPOC1 expression is upregulated as early as 8 h after Dox treatment, but a much stronger effect is visible after 16 h [4]. A time delay of SPOC1 expression could explain the observed extenuated effects. Nevertheless, based on the results gained from the two different experimental systems, we can conclude that this newly described antiviral effect of SPOC1 is MOI-independent and applies for different HCMV strains, as the same repressive effects were observed for the two HCMV strains TB40/E and AD169.

For adenoviral (Ad) infection, Schreiner and colleagues showed that SPOC1 leads to impaired viral DNA synthesis and the repression of viral transcription [2]. Also, for HIV-1, it is described that SPOC1 suppresses viral gene expression [3]. Consistent with these results, we found that viral transcription of HCMV is profoundly diminished when SPOC1 is expressed (Figure 4), which is also reflected at the protein level (Figure 3). Most interestingly, although we detected an undisturbed IE phase upon infection at high MOIs, we observed diminished transcription of IE genes at later time points of infection. We assume that SPOC1 exerts broad negative effects on HCMV transcription, most probably via association with viral genes of all temporal classes. Undisturbed IE gene expression at the onset of viral replication could be due to transient neutralization of SPOC1-mediated repression by components of the HCMV virion that are imported by infection and thus abundantly present under conditions of high MOI. Antagonization of SPOC1 by structural proteins has been reported for human adenovirus type 5, where the major core protein pVII associates with this chromatin remodeling protein followed by proteasomal degradation [2]. In contrast, no SPOC1 degradation could be observed during the first hours of HCMV infection; however, this does not exclude the possibility of functional antagonization by a viral factor that remains to be identified [4].

To gain more information on the potential mechanisms of SPOC1-mediated repression, we investigated its localization during the time course of lytic HCMV replication. During adenovirus infection, SPOC1 was shown to localize to viral replication centers (VRCs) and to directly interact with E2A-DBP, the adenovirus DNA-binding protein and a marker of VRCs [2]. When investigating SPOC1 localization at 48 h post HCMV infection, we observed a co-localization of SPOC1 with pUL44, a marker for VRCs (Figure 5A). The presence of SPOC1 in VRCs could indicate a possible direct interference with viral DNA replication or replication-specific factors, such as pUL44. pUL44 is the processivity factor of the viral DNA polymerase and important for proper viral replication [39]. In SPOC1-expressing cells, we detected a downregulation of UL44 both at the transcriptional and translational level (Figure 3 and Figure 4). The question of whether SPOC1 directly interferes with viral DNA replication remains to be answered. So far, we can state that viral factors required for efficient DNA replication are expressed at lower levels than normal when SPOC1 is present.

As the infection progresses, the exclusive co-localization with VRCs is lost, and we observe an accumulation of SPOC1 in a punctuate pattern at the rim of the nucleus (Figure 5A). Interestingly, pUL44 could also be detected in clusters outside of VRCs at later times of infection. As shown by click chemistry in combination with antibody staining, these accumulations of pUL44 co-localize with viral DNA and are found in close proximity to SPOC1 (Figure 5B). It is worth mentioning that pUL44 has been proposed to be not only important for viral DNA replication but also for efficient late viral gene expression [40]. Therefore, one may speculate that the observed re-localization of pUL44 to punctuate structures containing viral DNA indicates an association with sites of active late transcription.

One other essential protein for viral transcription is the cellular RNA polymerase II (RNAP II). Tamrakar et al. showed that phosphorylation of the C-terminal domain (CTD) of RNAP II, which regulates the mode of action of this protein, is finely tuned during HCMV infection. They also investigated the localization of the different phosphorylated isoforms of RNAP II during the replication cycle and found that, specifically, RNAP II S5P is located outside of VRCs at the nuclear rim during later times of infection [38]. They hypothesized that RNAP II S5P serves as a marker protein for regions where viral transcription takes place and that this event is physically separated from viral replication. Intriguingly, SPOC1 and especially the serine 5 phosphorylated form of RNAP II are known interaction partners [9]. When we investigated the localization of SPOC1 and RNAP II S5P during late time points of infection, we observed a co-localization of both proteins (Figure 8). Fuchs et al. reported that SPOC1 not only interacts with RNAP II S5P but also with subunits of the polycomb repressive complex 2 (PRC2), a well-known chromatin repressor. SPOC1 appears to stabilize the association of both factors with the genome and thereby exerts a regulating role in the transcriptional level [9].

Specifically, the combination of SPOC1, PRC2, RNAP II S5P, H3K4me2/3 and H3K27me3 was found to be predominantly associated with repressed genes [10]. Intriguingly, when we stained infected HFF/SPOC1 with antibodies against SUZ12 and EZH2 we found a clear co-localization of SPOC1 and the components of the PCR2 complex, again close to viral DNA. SUZ12 is supposed to serve as the interacting factor of SPOC1 and the PRC2 complex [10]. In co-immunoprecipitation (CoIP) analyses, we detected a clear interaction of SPOC1 and EZH2 (Figure 7). Although we cannot exclude an indirect interaction potentially mediated by other proteins of the PRC2 complex or via DNA bridging, the CoIP indicates that the N-terminal domain of SPOC1 is crucial for the interaction of PRC2 and SPOC1.

Taken together, we found SPOC1 to localize first in viral replication centers and later, additionally, in punctuate clusters at the nuclear rim and outside of VRCs, where it co-localized with RNAP II S5P and PRC2 components in close proximity to viral DNA. Since we observed that SPOC1 expression leads to a downregulation of a number of viral genes at the mRNA level, we propose that SPOC1, a known chromatin reader and transcriptional co-regulator, represses viral genes utilizing repressive cellular factors such as PRC2, and this takes place at specific nuclear sites outside of VRCs. Due to inefficient generation of viral gene products, viral DNA replication and virus release are impaired. Since, in contrast to results obtained at low MOI, the enzymatic PRC2 inhibitor GSK126 only marginally affected H3K27me3 levels at high MOI, further experiments will be necessary to clarify the contribution of PRC2-mediated histone modification to the repression of viral genes [37].

Lastly, we would like to highlight the fact that SPOC1 was found to be decreased in a GSK-3β-dependent manner during later times of HCMV infection [4]. Downregulation of SPOC1 has also been detected during HIV-1 infection, which is mediated by the viral protein Vpr and probably counteracts the antiviral effects of SPOC1 against HIV-1 after integration [14]. The GSK-3β-dependent degradation of SPOC1 observed during HCMV infection may therefore represent a viral countermeasure of the repressive effects of SPOC1 on late viral transcription.

## Data Availability

Data are contained within the article and Supplementary material.

## Supplementary Materials

The following supporting information can be downloaded at: https://www.mdpi.com/article/10.3390/v16030363/s1, Figure S1: HFF/mCherry cells during HCMV infection, Figure S2: Overview picture of HFF/SPOC1 infected with AD169, MOI 1. Table S1: Oligonucleotides and sequences
