# Supplementary material for: Discovery of a Novel Antiviral Effect of the Restriction Factor SPOC1 against Human Cytomegalovirus
Source: Viruses. 2024 Feb 27;16(3):363. doi: 10.3390/v16030363 (PMC10976249; doi:10.3390/v16030363)
Supplement: Supplementary file 1 [file viruses-16-00363-s001.zip › viruses-2813357-supplementary.pdf]

Table S1*: Oligonucleotides and sequences*

All used primers were purchased from Biomers GmbH (Ulm, Germany) if not indicated otherwise.

**Oligonucleotides for cloning**
SPOC1 WT (aa 1-300)
3'CATAGAATTCTCAGTCCAGGAACAGCTTCC
5'CATAGGATCCGACTCTGACTCTTGCGC
SPOC1 aa 1-231
3'CATAGAATTCTCAGTCCCAGGAATCGTCATCT
SPOC1 aa 1-150
3'CATAGAATTCTCACTCCACGTAGGGGTCAG
**Oligonucleotides for qRT-PCR (TaqMan)**
CMV/IE1
3'GAGCAGACTCTCAGAGGATCGG
5'AAGCGGCCTCTGATAACCAAG
MIE FAM/TAMRA<br>CATGCAGATCTCCTCAATGCGGCG
Albumin
3'GCATGGAAGGTGAATGTTTCAG
5'GTGAACAGGCGACCATGCT
Alb FAM/TAMRA<br>TCAGCTCTGGAAGTCGATGAAACATACGTTC
**Oligonucleotides for qRT-PCR (SYBR)**
**IE1,** see Oligonucleotides for qRT-PCR (TaqMan), *CMV/IE1*
GAPDH Real Time PCR Primer Set (VHPS-3541, Biomol GmbH)
UL122 (IE2) [24]
3'TGACCGAGGATTGCAACGA
5'CGGCATGATTGACAGCCTG
**US3** [25]
3'TGTTTCTCGGTGAAGTTGCC
5'CTGGATGTGGTGGTATCGGA
UL44 [25]
3'ACGCGTAATTCACCACGGGCA
5'TGTGGTCATTGTGCCCGCCC
UL82 (pp71) [26]
3'ACGACACCGTAGACCTGACC
5'AAAGAGGTGCAGTCCGCTAA
UL86 (MCP) [25]
3'ACCTCGAAGGTGTCGGTGCGT
5'CCAAGGCGCACATCCACCCG
UL99 (pp28) [27]
3'CTTTGCTGATGGTGGTGATG
5' GAGGACAAGGCTCCGAAAC

Figure S1

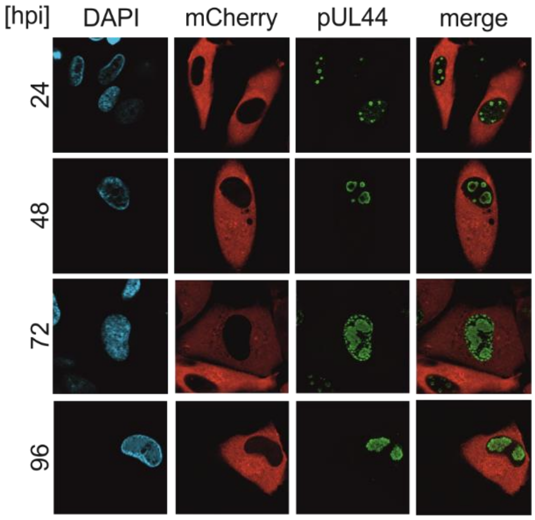

Figure S1: HFF/mCherry cells during HCMV infection. Cells were infected with AD169, MOI 1 and fixed at indicated time points. An antibody against pUL44 was utilized in combination with secondary antibody Alexa 488. DAPI was used to visualize the cell nuclei.

Figure S2

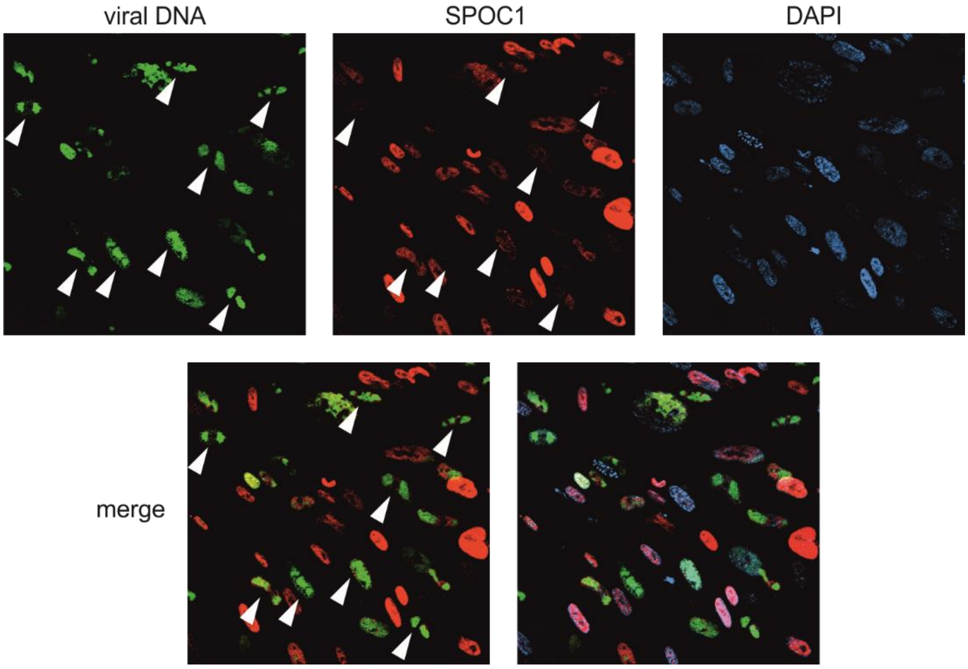

Figure S2: Overview picture of HFF/SPOC1 infected with AD169, MOI 1. 72 hpi EdC was added to the cells prior to fixation at 96 hpi. Viral DNA was visualized by click chemistry. Additionally, the samples were treated with an antibody against SPOC1 in combination with Alexa-555. DAPI staining was used for visualization of cell nuclei. Arrows indicate cells with large and intense viral replication centers.
